# Study on Cynodon dactylon root system affecting dry–wet cracking behavior and shear strength characteristics of expansive soil

**DOI:** 10.1038/s41598-023-39770-7

**Published:** 2023-08-11

**Authors:** Yingzi Xu, Yanyan Guo, Zhen Huang, Dezhi Liu, Quanen Huang, Hong Tang

**Affiliations:** 1 School of Civil Engineering and Architecture, Guangxi University, Nanning, 530004 China; 2 Key Laboratory of Disaster Prevention and Structural Safety, Guangxi University, Nanning, 530004 China

**Keywords:** Civil engineering, Environmental sciences, Natural hazards

## Abstract

Expansive soil exhibits remarkable characteristics of water absorption expansion and water loss shrinkage, rendering it susceptible to cracking under the alternating dry–wet environments of nature. The generation and development of cracks in expansive soil can result in catastrophic engineering accidents such as landslides. Vegetation protection is an important approach to stabilizing expansive soil slopes and fulfilling ecological protection requirements. In this study, through indoor experiments and theoretical analysis methods, the effects of Cynodon dactylon roots on the crack development and shear strength of expansive soil subjected to dry–wet cycles were analyzed, and the relationship between the crack development and shear strength decay in root-doped expansive soil was explored. Furthermore, the mechanism of vegetative root system action was elucidated. The results show that the Cynodon dactylon root system exerts a significant inhibitory effect on crack development in expansive soil. The crack indexes of root-doped expansive soil exhibit significant phase characteristics during the process of dry–wet cycles. The crack-blocking and reinforcing effect of the root system becomes pronounced as the root-to-soil mass ratio increases and the root diameter decreased. Moreover, the process of crack development in expansive soil is accompanied by a decrease in soil shear strength. The quantitative relationship between crack development and shear strength decay can serve as a basis for predicting the stability of slope soil. Overall, the results highlight the potential of vegetation-based approaches in protecting slopes with expansive soils and have practical implications for ecological protection and engineering design in areas with expansive soils.

## Introduction

Expansive soil is a highly plastic clay with characteristics of cracking, expansion and contraction, and super consolidation. Due to the richness of hydrophilic minerals such as montmorillonite, the change in water content will cause significant wet expansion and dry contraction deformation of the soil, which will lead to the generation of cracks^1,2^. The repetitive cycles of rainfall and evaporation further exacerbate the cracking process, deteriorating the overall soil structure. Consequently, the strength of the expansive soil is compromised, posing various geotechnical challenges such as slope landslides and road cracking^3–5^. The preservation of long-term stability in expansive soil slopes has emerged as a critical concern in the field of engineering, necessitating effective solutions to address this issue.

Scholars both domestic and international have accumulated significant expertise in the reinforcement of expansive soil slopes through extensive practical exploration. Various rigid and flexible protection measures have been actively adopted in engineering practice to mitigate the risks associated with expansive soil slopes^6–9^. However, the durability and economic efficiency of traditional methods of support have been suboptimal, prompting researchers to explore alternative approaches such as incorporating fiber^10–12^, lime^13–15^, and fly ash^16,17^ into the soil.

In recent years, there has been a growing interest in the technical and economic advantages, as well as the ecological benefits, of using ecological slope protection methods. The concept of “treating expansion with flexibility and green protection” has emerged as a novel approach to expansive soil protection management^5,18^. These studies have demonstrated the reinforcing effect and ecological restoration potential of environmental protection measures on slope engineering^19^, and have emphasized the close relationship between the root system and soil cracks^20–24^, as well as the strong correlation between root growth characteristics and slope stability^25,26^. However, despite these significant findings, the influence of vegetation roots on the development of expansive soil cracks has not been fully addressed in the existing literature, and there is a lack of discussion of the impact of root growth characteristics on the development of expansive soil cracks.

The shear strength decay in expansive soils due to crack development has been extensively investigated in recent studies^27–29^. Dry–wet cycles have been identified as a significant factor contributing to the development of cracks in expansive soils^30–32^, leading to a decay in soil strength. On the other hand, the presence of vegetation roots has been found to enhance soil shear strength to some extent^33–35^. However, the inhibitory effect of the root system on the shear strength decay of expansive soil and the relationship between crack development and shear strength decay remains unclear.

Building upon the existing studies, this study analyzed the effects of characteristic parameters of the Cynodon dactylon root system on the dry–wet cracking behavior and shear strength of expansive soil through controlled indoor tests. The study also explored the relationship between the crack development process and shear strength decay in root-doped expansive soil and elucidated the mechanism of Cynodon dactylon roots to enhance the performance of expansive soil. The research results can provide a more effective theoretical basis for the development and application of slope vegetation ecological treatment technology in expansive soil areas, and also provide a useful reference for the study of landslides and other geological hazards in expansive soil areas.

## Materials and methods

### Materials

**Figure 1:**
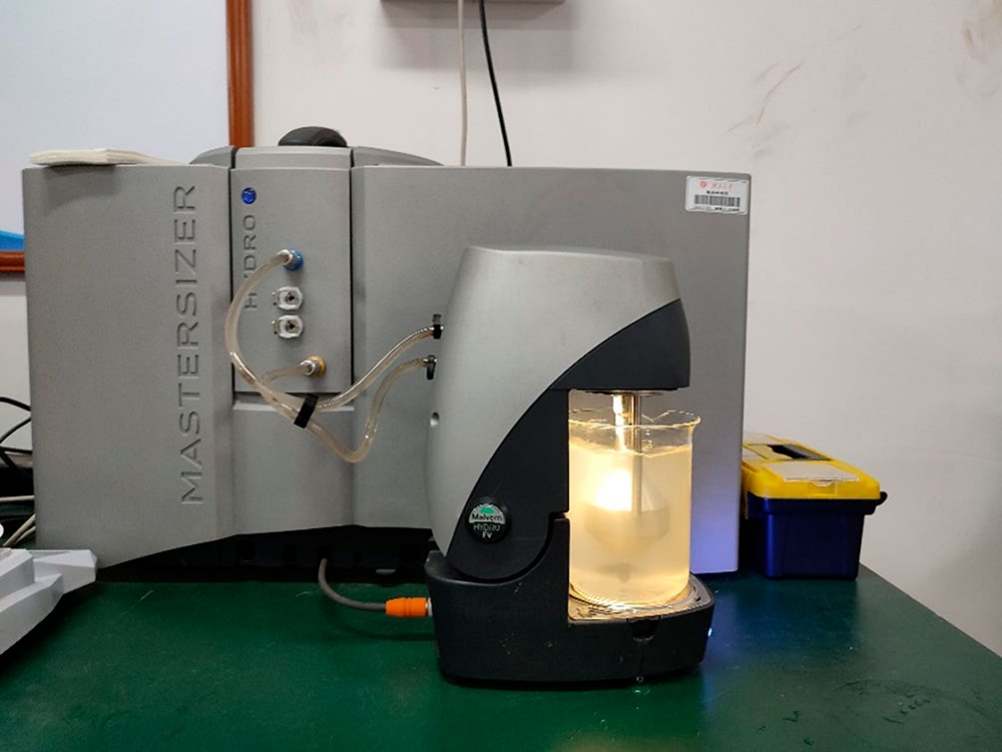
MASTERSIZER 3000 (MAZ 3000) laser particle size analyzer.

**Figure 2:**
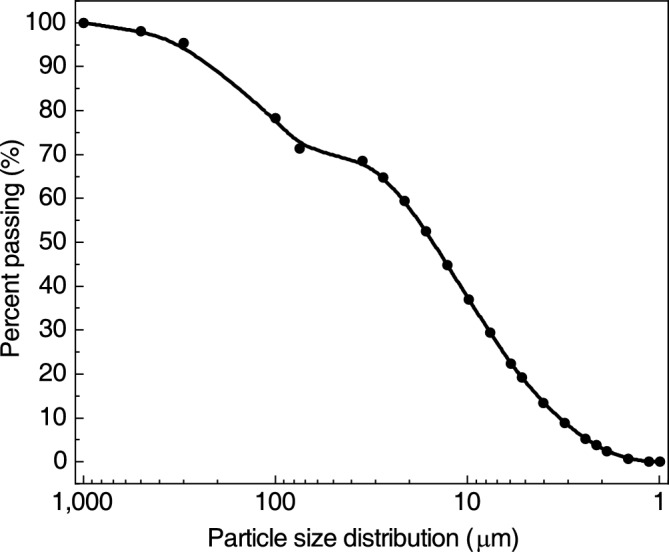
Particle size distribution of expansive soils.

**Figure 3:**
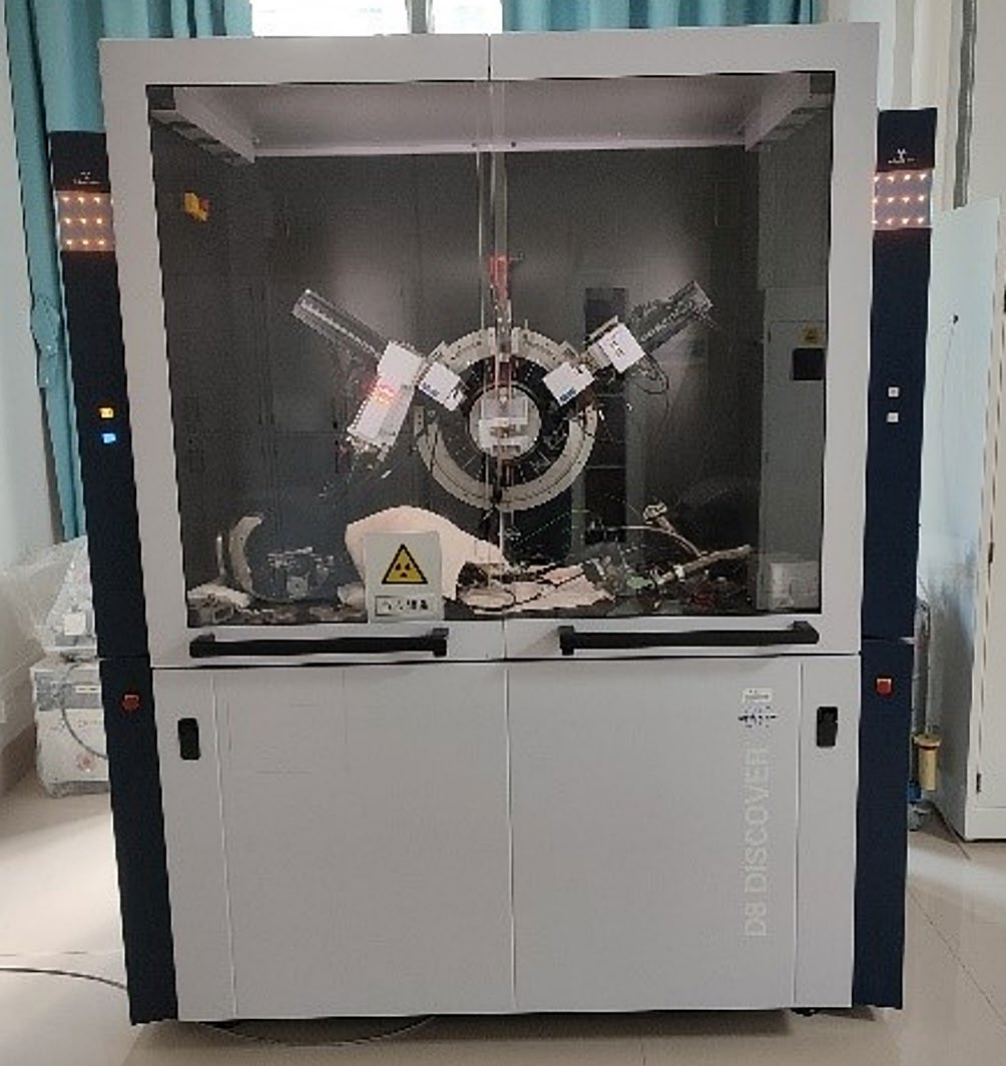
Bruker XRD Goniometer (D8 DISCOVER).

The soil samples used in this study were taken from the on-site slopes of expansive soils located in Xingning District, Nanning City, Guangxi Autonomous Region, China. The basic physical and mechanical properties of the soil samples are listed in Table 1. The soil used for the test was determined as medium expansive soil, following the domestic “Technical Regulations for Construction in Expansive Soil Areas” (GB 50,112–2013). The particle size distribution of the expansive soil was determined using a combined laser particle size meter MASTERSIZER 3000 (MAZ 3000) from Malvern Instruments Ltd, UK, and a sieving method, as shown in Fig. 1. The sieving method is mainly used for expansive soil specimens with a particle size above 1 mm. After sieving, soil samples with a particle size below 1 mm are used in the MAZ 3000 laser particle sizer for further particle size analysis. The particle size distribution of the expansive soil is organized as shown in Fig. 2. The mineralogical composition of the soil samples was determined using a Bruker XRD Goniometer (D8 DISCOVER) made in Germany and the MDI JADE 6 software was used for the physical phase retrieval and analysis, as shown in Fig. 3. The mineral composition of the expansive soils is organized as shown in Table 2.

**Table 1:** Basic physical and mechanical properties of soil samples.

Natural water content (%)	Natural dry density (g/cm^3^)	Optimum water content (%)	Maximum dry density (g/cm^3^)	Liquid limit (%)	Plastic limit (%)	Plasticity index (%)	Free expansion rate (%)
23.3	1.41	19.0	1.72	48.6	21.6	27	65

**Table 2:** Mineral composition and content of the expansive soils.

Mineral content (%)						
Montmorillonite	Elysium	Kaolinite	Chlorite	Feldspar	Quartz	Mica
16	20	26	2	1	33	2

**Figure 4:**
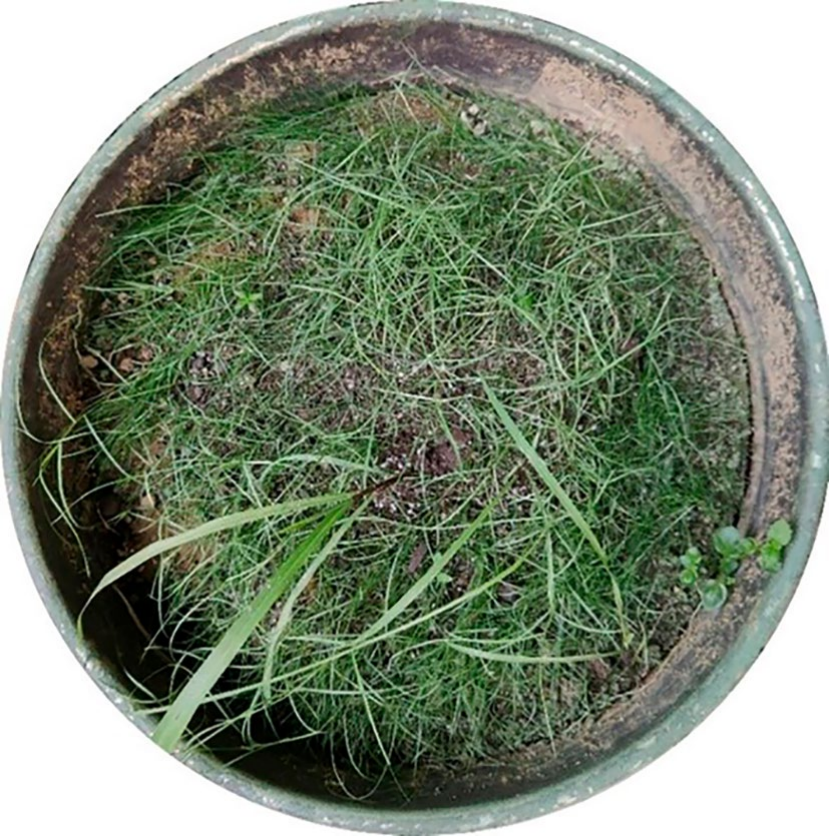
Cynodon dactylon planting.

**Figure 5:**
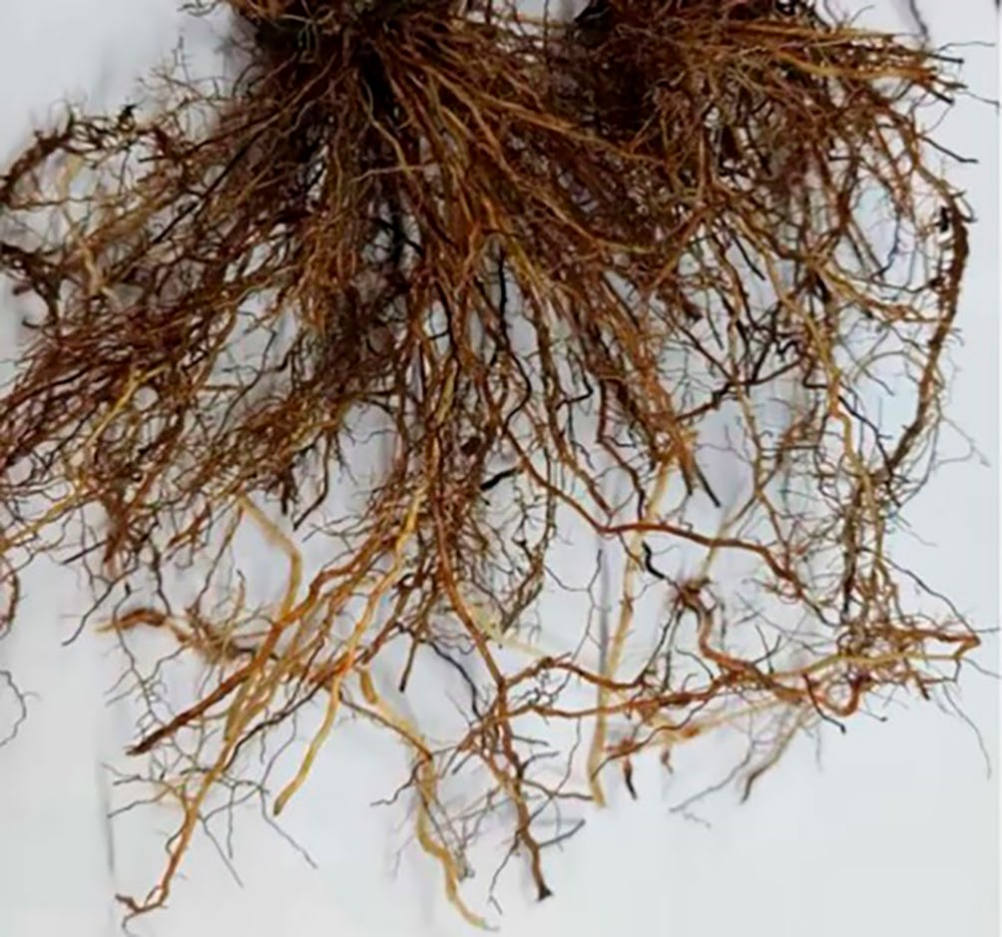
Cynodon dactylon roots.

The plant root system selected for the study was the Cynodon dactylon roots system. Cynodon dactylon is a fibrous-rooted plant with diminutive and tenacious fibrous roots. The Cynodon dactylon roots used in this study were sourced from indoor cultivation and are not wild sources or rare preserved genetic resources. As shown in Fig. 4, Cynodon dactylon was planted in an iron bucket of 60 cm diameter and 60 cm height with regular watering and fertilization for 3 months. The root system of Cynodon dactylon is shown in Fig. 5. Cynodon dactylon seeds were produced in large quantities by major commercial seed producers and purchased from local markets in China, and plants were grown according to relevant guidelines. Cynodon dactylon is extensively distributed in tropical and subtropical regions, and also grows in temperate areas, rendering it become one of the best plant species for ecological slope grass^36,37^. The Cynodon dactylon roots grow mostly within the soil depth range of 0–40 cm with a well-developed root system and minimal disparities in root diameter per 10 cm depth interval^38,39^. Consequently, it serves as an appropriate specimen for laboratory experiments.

### Test program

To investigate the effects of root-to-soil mass ratio and root diameter on the development of cracks in expansive soil, these two influencing factors were tested individually and in combination. Following the collection of Cynodon dactylon roots from the test site, the roots were thoroughly washed with water and subsequently weighed after removing surface water using absorbent paper. To minimize the influence of root length in experimental results, the root system was uniformly cut to a length of 2 cm, based on the ring knife sample size (61.8 mm in diameter and 20 mm in height) used in indoor tests. To replicate the natural growth conditions of Cynodon dactylon roots in the soil, the soil and roots were mixed well and evenly.

The determination of soil sample compactness in this indoor test was based on the actual compactness of the slope on-site and resulted in a value of 85%. Experimental environment temperatures of 25 ℃ and 40 ℃ were selected considering the local temperature change conditions. Based on the previous slope protection vegetation planting tests and statistical analysis of the root system^38,40^, the root-to-soil mass ratio was set at 0%, 0.06%, 0.14%, and 0.22%, and the root diameter was set to 0.0–0.4 mm, 0.4–0.6 mm, and 0.6–0.8 mm.

**Figure 6:**
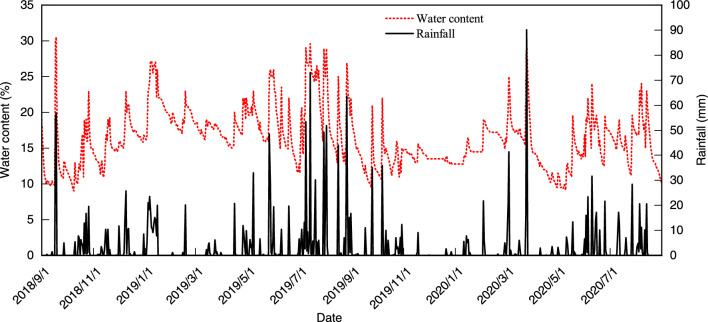
Time course curve of soil water content and rainfall variation.

During the period of monitoring, the water content of the soil exhibited a range of values, with the minimum recorded at 9% and the maximum at 30.5%, as shown in Fig. 6. By integrating the actual monitoring data with the optimal water content of the soil sample (19%), the initial water content for the equal-amplitude dry–wet cycle test was determined to be 19%. The minimum and maximum water contents for the dry–wet cycle were established at 9% and 29% respectively, the dry–wet cycle amplitude was ± 10%, and the dry–wet cycles were 6 times.

Table 3 presents the test program employed in this study. Test 1 aimed at exploring the effect of the root system on crack development at a temperature of 25 °C. Notably, the influence of temperature was found to promote earlier and more rapid crack development and stabilization, without significant effects on the final index^41^. To speed up the experimental process, Tests 2–7 were conducted at a higher temperature of 40 °C. Specifically, Tests 2–5 were carried out to investigate the effects of root-to-soil mass ratio on crack development and soil shear strength, while Tests 5–7 were conducted to examine the effects of root diameter on crack development and soil shear strength.

**Table 3:** Experimental protocol.

Number	Number of soil samples	Temperature (℃)	Root-to-soil mass ratio (%)	Root diameter (mm)
1	4	25	0.14	0.0–0.4
2	4	40	0	0
3	4	40	0.06	0.0–0.4
4	4	40	0.14	0.0–0.4
5	4	40	0.22	0.0–0.4
6	4	40	0.22	0.4–0.6
7	4	40	0.22	0.6–0.8

### Test process

**Figure 7:**
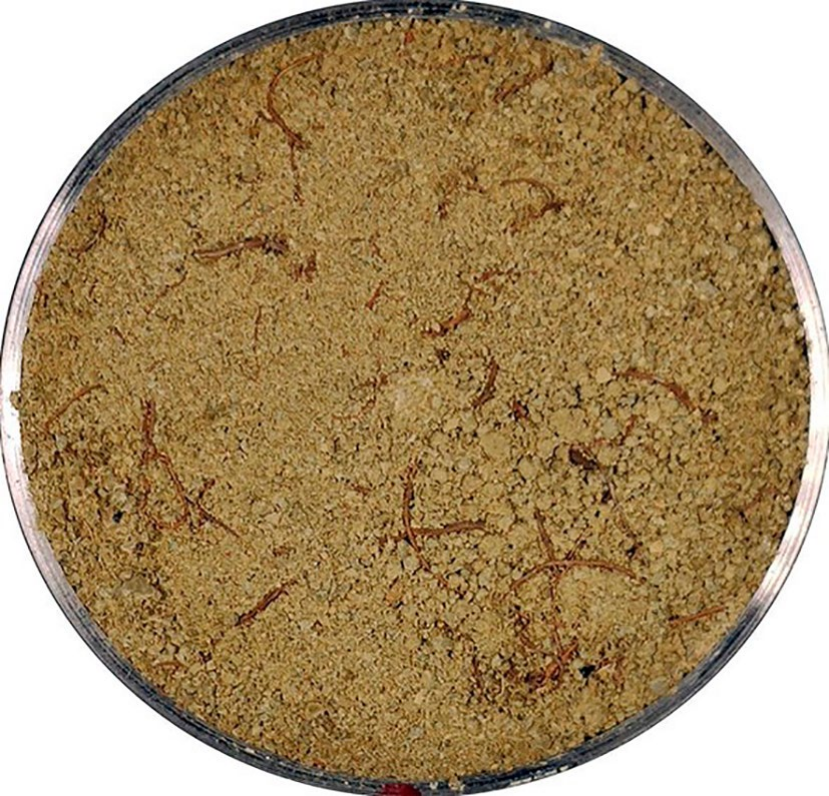
Soil sample.

The expanded soil underwent the following preparation process. Initially, the soil was air-dried and subsequently crushed before passing through a 2 mm sieve to ensure uniformity. The prepared soil material was then accurately weighed and spread in layers in an enamel tray. To achieve the desired water content of 19%, the surface of the soil was evenly sprayed with distilled water and thoroughly mixed. The resulting soil sample was then hermetically sealed for 24 h to allow for complete water diffusion. When the stewing was completed, the soil sample was mixed with the roots of Cynodon dactylon fully and uniformly. The compaction of the soil sample was controlled by regulating the quality of the soil sample, and the static pressure method was employed to make the sample at one-time, as shown in Fig. 7. Notably, before the soil sample preparation, petroleum jelly was meticulously applied to the inner wall of the ring knife and the contact area between the iron mat and the soil sample surface to mitigate potential boundary effects during the subsequent dry–wet cycle tests.

The dry–wet cycle process was divided into two steps humidification and dehumidification. To ensure precise control over water content, an electronic balance with an accuracy of 0.01 g was employed. For the humidification process, the soil sample was placed in an enamel tray, and a single sheet of wet filter paper was applied to both the top and bottom surfaces. This was done to prevent soil sample disintegration upon water absorption and to ensure uniform wetting. A small spray was used to spray water on the filter paper on the top surface of the soil sample to bring the mass to be weighed to a preset target water content. The dehumidification process was carried out in a constant temperature test chamber at 25 °C and an oven at 40 °C. The soil samples were weighed and photographed several times during this process, and the dehumidification was considered to be completed when the mass of the sample changed within 0.02 g. This constituted 1 dry–wet cycle, and the process was repeated for 0–6 cycles.

**Figure 8:**
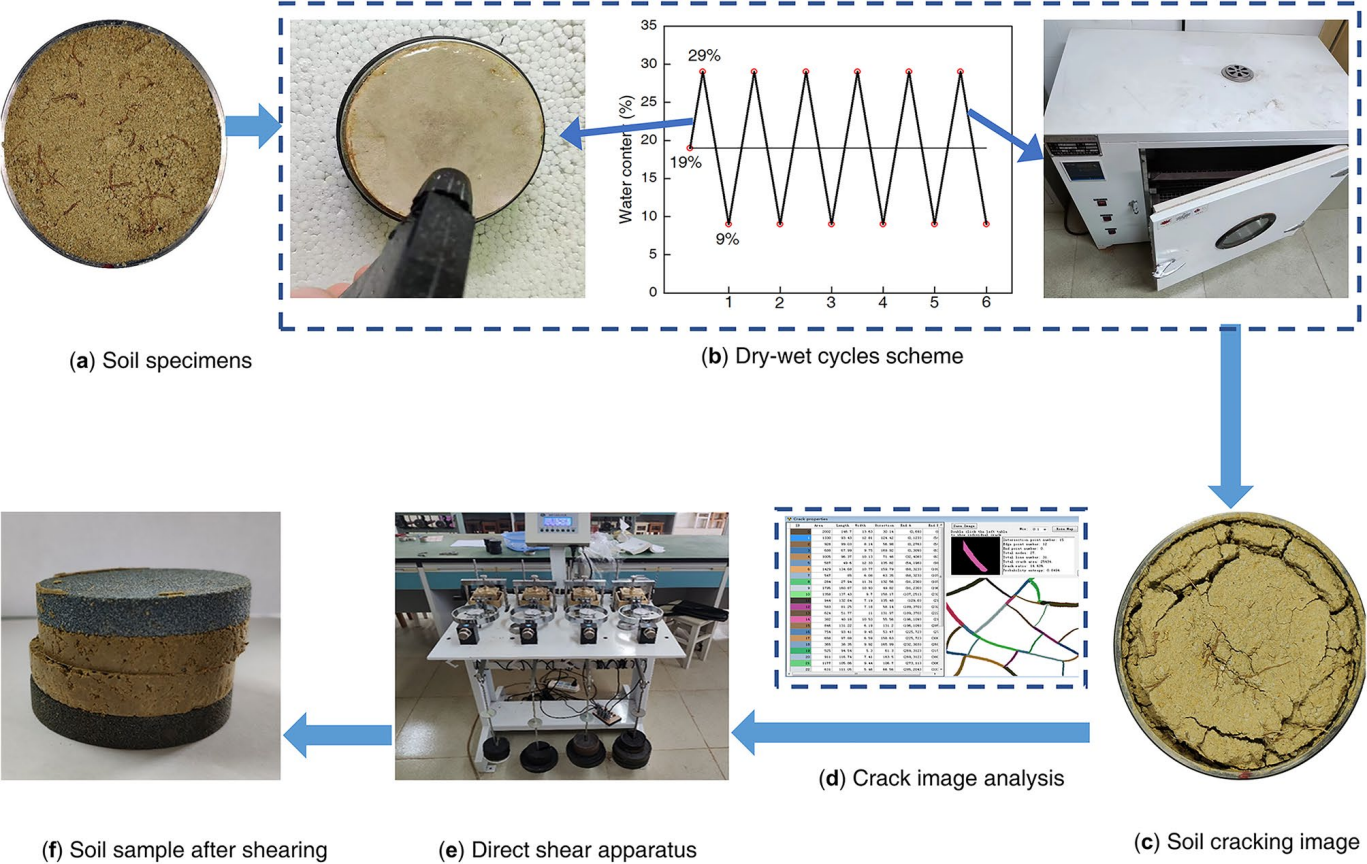
Dry–wet cycle test and straight shear test process.

Direct shear tests were conducted on soil samples collected at the end of each dry–wet cycle test. The undrained shear test was conducted following the domestic “Highway Geotechnical Test Procedure” (JTG 3430–2020). Four replicates of soil samples were prepared for each test group, and the vertical pressure was applied at levels of 100, 200, 300, and 400 kPa, while the shear rate was maintained at 0.8 mm/min. A straight line is formed with the vertical pressure as the horizontal coordinate and the shear strength as the vertical coordinate. The inclination angle of this line is the angle of internal friction, and the intercept point on the vertical coordinate is the cohesive force. Where the shear strength is obtained from the peak point of the relational curve with shear stress as the vertical coordinate and shear displacement as the horizontal coordinate, the shear stress is calculated from the correction factor of the dynamometer in the shear test, the reading of the dynamometer and the initial area of the specimen. The experimental setup and methodology are shown in Fig. 8.

To ensure consistent light conditions, only the same light source in the room was used to take pictures with equal focus (20 cm). Subsequently, the acquired crack images were processed using Adobe Photoshop software and imported into PCAS^42,43^ software, a proprietary tool developed by Nanjing University, for a series of pre-processing steps such as automatic binarization, noise removal, and skeletonization. Based on the obtained crack binary diagram and its skeleton, the soil crack development pattern was quantitatively characterized by the statistical soil sample crack rate, average crack width, and other indicators. The crack rate was defined as the ratio of the surface crack area to the total area of the soil sample, characterizing the overall development of the crack of the soil sample. To ensure consistency in crack rate calculation, the upper surface area of the ring knife was used as the total area in the test. The number of cracks was determined based on the definition of a crack trace between two adjacent nodes as one crack. The average crack length was calculated as the ratio of the total length of cracks to the number of cracks. The average crack width was calculated as the ratio of the total width of cracks to the number of cracks. The connectivity was defined as the ratio of the number of crack intersection points (A) to the sum of the number of intersection points (A) and endpoints (B), expressed as A/(A + B).

### Ethical statements

The Cynodon dactylon roots used in this study were sourced from indoor cultivation and are not wild sources or rare preserved genetic resources. Plant seeds are produced in large quantities by major commercial seed producers and can be purchased from local markets in China. All plant experiments were carried out following relevant guidelines.

## Results and discussion

### Expansive soil cracking behavior

#### Effect of root growth characteristics on dehiscence behavior

**Figure 9:**
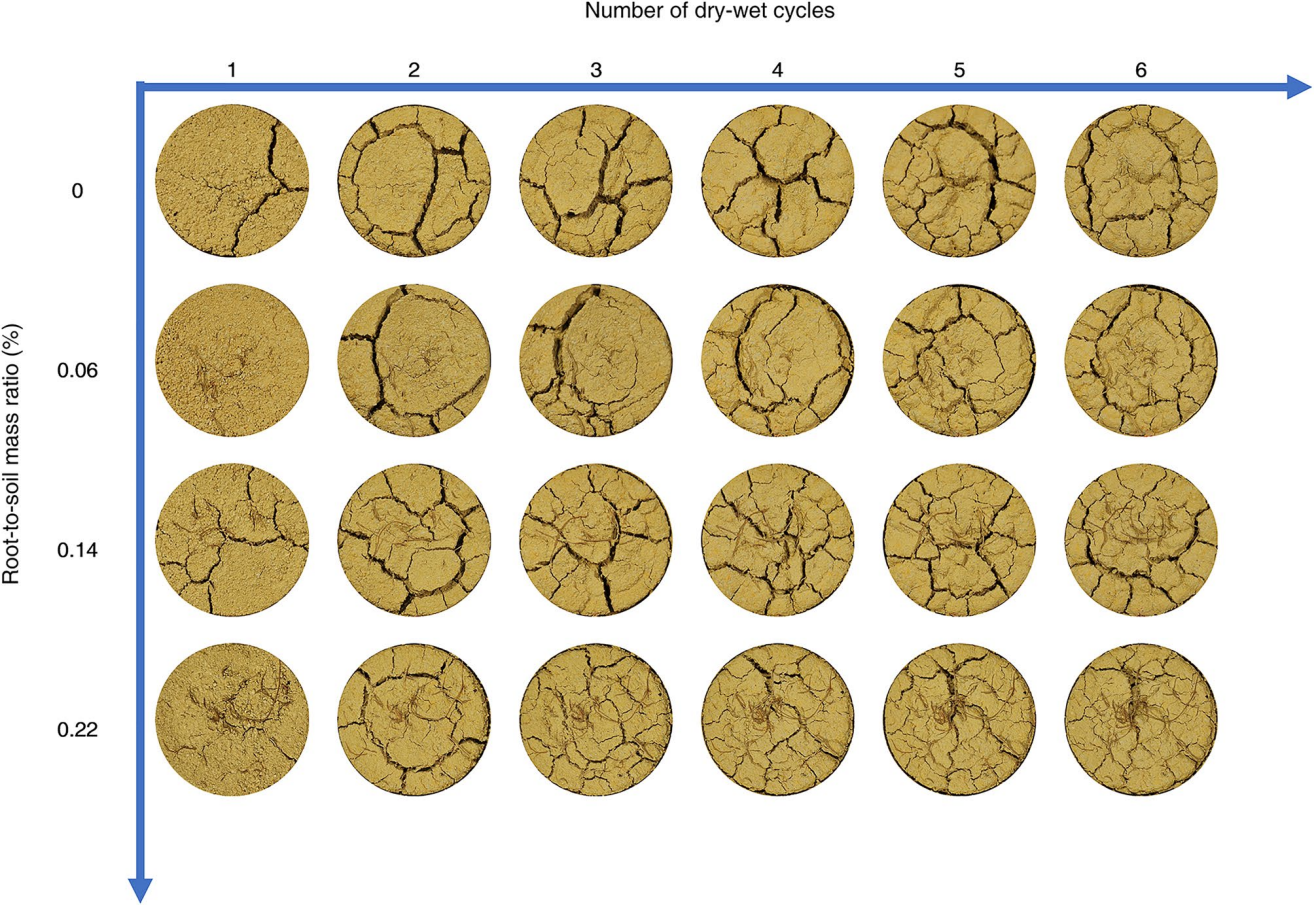
Crack network images of expansive soils (different root-to-soil mass ratios).

**Figure 10:**
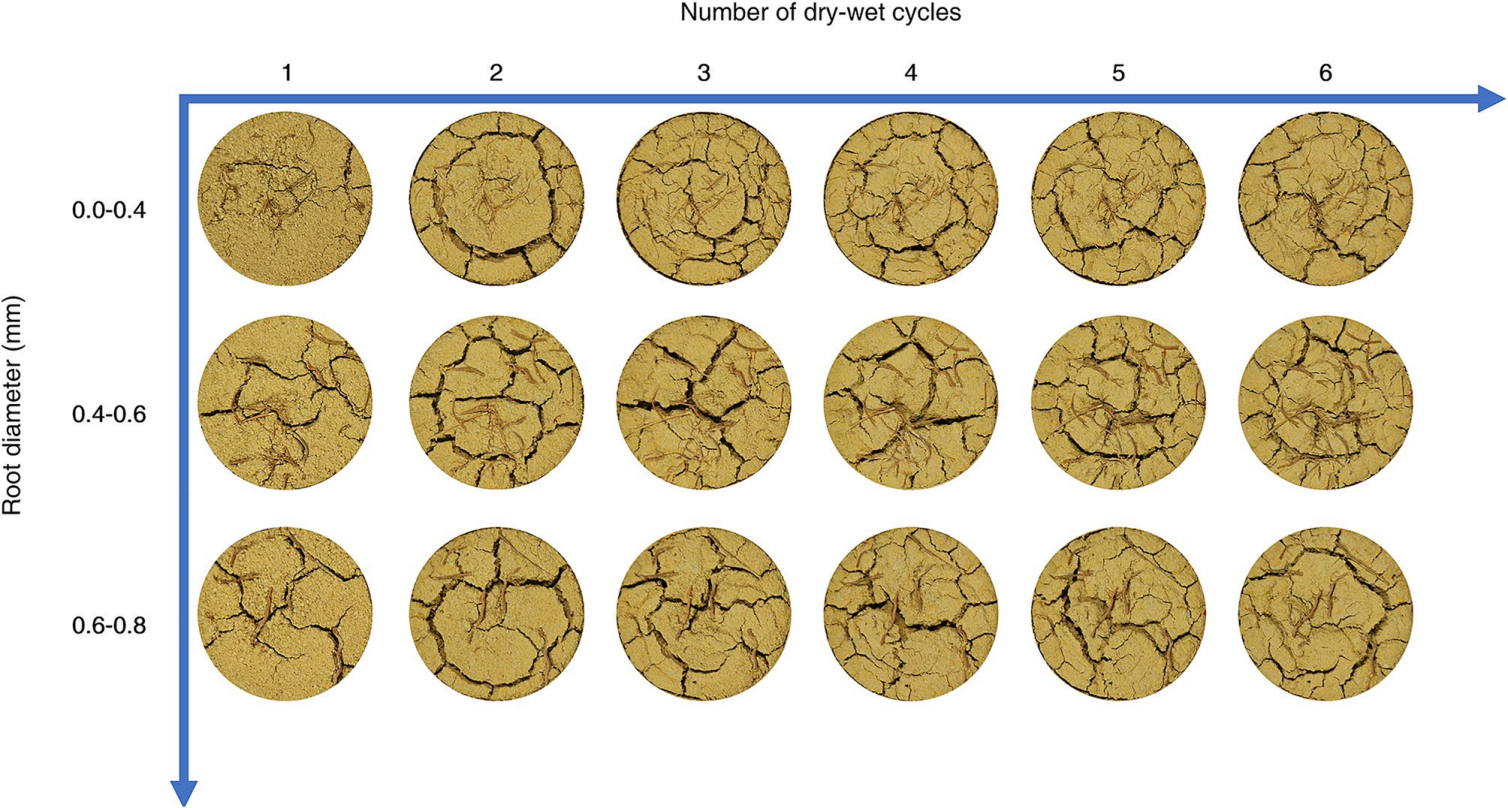
Image of the crack network of expansive soil (different root diameters).

Figure 9 shows the crack network images of the expansive soil samples with different root-to-soil mass ratios after each dry–wet cycle at 40 °C and 0–0.4 mm root diameter. Figure 10 shows the crack network images of expansive soil samples with different root diameters after each dry–wet cycle at 40 °C and 0.22% root-to-soil mass ratio. It can be seen that coarse and long penetration cracks appeared on the surface of the pure expansive soil from the 1st cycle. With repeated drying-wetting cycles, the cracks gradually extended toward the middle of the soil samples and eventually, the main cracks divided the surface into several large areas. This is mainly due to the abundance of clay minerals, such as montmorillonite, in expansive soils. The surface of clay particles is wrapped by a hydrated film due to their strong hydrophilicity^44^, which provides space for water loss and shrinkage of expansive soils and is a prerequisite for the development of expansive soil cracks. During the drying process of expansive soil, the water gradually evaporates, and the soil particles will close to each other in the horizontal direction and consolidate in the vertical direction under the action of surface tension and suction, which is manifested as the volume contraction of the soil in the macroscopic direction. Surface tension and suction forces cause each soil particle in the soil to be subjected to tensile stress in the horizontal direction, thus creating a tension stress field. When the magnitude of the tensile stress field exceeds the tensile strength of the soil, cracks are created^45^. Water exchange is more frequent at the edge of the soil sample, so it is more likely to produce fissures, so the fissures are gradually approaching from the edge to the middle. The healing of cracks at the edge of the 6th dry–wet cycle can be attributed to two possible reasons. Firstly, the wider cracks cause the collapse and subsequent filling of soil particles at the crack edges. Secondly, the reversal of the water content gradient causes shrinkage in the upper cracks.

Furthermore, when root-doped expansive soils underwent dry–wet cycles, cracks also expanded towards the middle of the soil but were more numerous and finer compared to pure expansive soils. This phenomenon becomes more pronounced with increasing root-to-soil mass ratio and decreasing root diameter. The cracks at the “bridging” position of the root system exhibited obvious healing, as evidenced by the recovery of cracks in the middle of the soil samples with root-to-soil mass ratios of 0.06% and 0.14%, as well as root diameters ranging from 0.4 to 0.6 mm and 0.6–0.8 mm.

**Figure 11:**
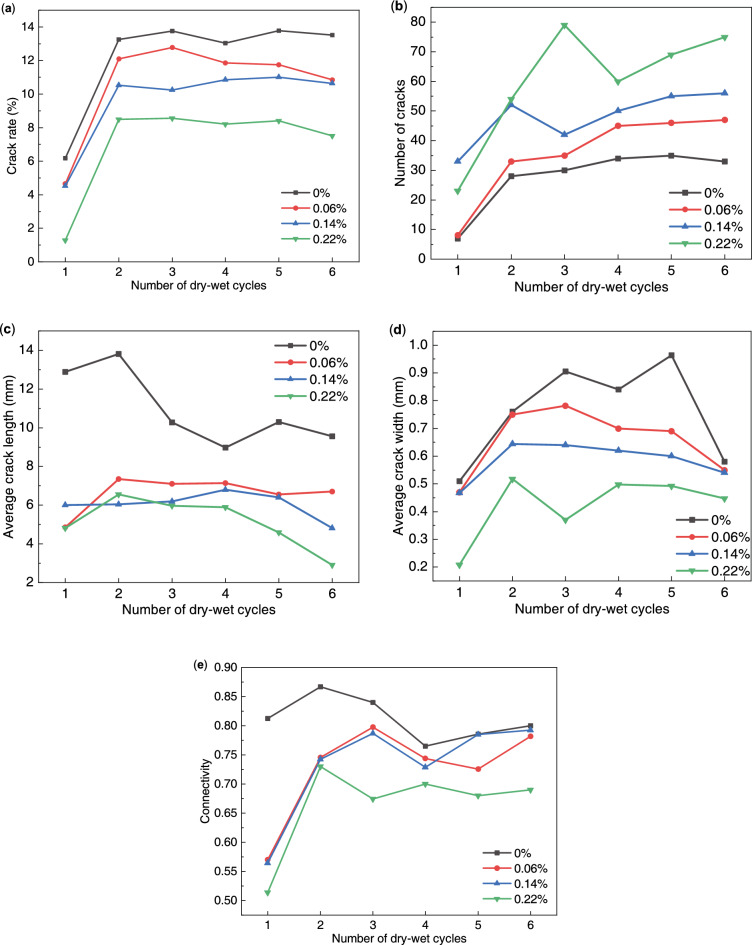
Variation curve of the crack index of expansive soil with the different root-to-soil mass ratios under the condition of dry–wet cycles: (**a**) crack rate; (**b**) number of cracks; (**c**) average crack length; (**d**) average crack width; and (**e)** connectivity.

Figure 11 shows the variation curves of the crack index of expansive soils with different root-to-soil mass ratios under the condition of dry–wet cycles. The crack rate of expansive soil exhibited a rapid increase during the 2nd dry–wet cycle and a slight decrease without significant fluctuations in subsequent cycles. The average width and the connectivity of cracks exhibited a trend of increasing and then decreasing during the dry–wet cycles. Notably, expansive soil samples with a root-to-soil mass ratio of 0.22% exhibited a significant reduction in the average width and the connectivity of cracks during the 3rd dry–wet cycle. Combined with Fig. 9, it can be seen that there was root distribution at the wide crack of the soil sample in the 2nd cycle, which caused a certain degree of healing of the soil crack in the process of humidification. Furthermore, the number of cracks in expansive soil exhibited an overall increasing trend with an increase in the number of dry–wet cycles, but there were large fluctuations in the soil samples with 0.14% and 0.22% root-to-soil mass ratio. The average crack length on the surface of expansive soils steadily increases in the early stages of the wet-dry cycle and tends to decrease in the later stages^46^. This is mainly because the surface of soil samples at the late stage of the dry–wet cycles are mostly small and disconnected cracks, the number of cracks increases significantly, and the average length of cracks decreases.

It can be seen from the indicators at the end of the 6th dry–wet cycle that the crack rate, the average crack width, connectivity, and the average crack length exhibited an overall decreasing trend with the increase of root-to-soil mass ratio. Remarkably, the number of cracks exhibited an increasing trend with the increase in the root-to-soil mass ratio, which can be attributed to the root system inducing inhomogeneity and anisotropy in the soil, thereby increasing the weak points on the soil surface. This also explains the phenomenon that the number of cracks in the root-doped soil samples is significantly higher than that of pure expansive soil during dry–wet cycles. Furthermore, the variation in the connectivity patterns indicates that soil samples with higher root-to-soil mass ratios predominantly exhibit fine and disconnected cracks on the surface. It reveals that the root crack-blocking effect of the root system is a positive correlation with the root-to-soil mass ratio. Notably, the optimal root crack-blocking effect was achieved at a root-to-soil mass ratio of 0.22%.

**Figure 12:**
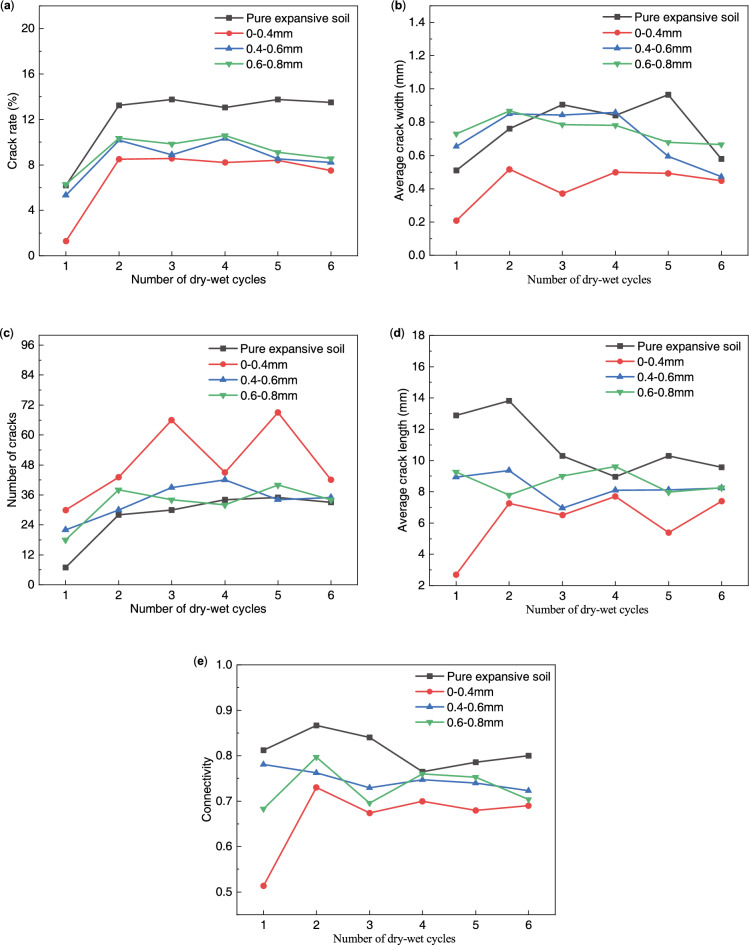
Variation curve of the crack index of root-doped expansive soil with different root diameters under the condition of dry–wet cycles: (**a**) crack rate; (**b**) average crack width; (**c**) number of cracks; (**d**) average crack length; and (**e)** connectivity.

Figure 12 shows the change curve of the crack index of expansive soil with different root diameters under the condition of dry–wet cycles. The crack rate of expansive soil exhibited a rapid increase during the 2nd dry–wet cycle and a slight decrease without significant fluctuations in subsequent cycles. The average crack width exhibited an overall trend of increasing 1st and then decreasing. The difference between the average width of the crack of pure expansive soil and expansive soil with 0.4–0.6 mm and 0.6–0.8 mm root diameter in the first four dry–wet cycles was not large, and it was reduced to different degrees in the 5th dry–wet cycle. The trend in average crack width corresponded with the trend in crack rate. The overall trend of the number of cracks was increasing. Specifically, the number of cracks in soil samples with a root diameter of 0.0–0.4 mm exhibited significant fluctuations, resulting in a distinct “bimodal” shape in the change curve. The average crack length and connectivity also exhibited substantial fluctuations. The connectivity of soil samples with root diameters of 0.0–0.4 mm initially increased and then decreased. In contrast, pure expansive soil and root diameter 0.4–0.6 mm soil samples exhibited an overall decreasing trend in connectivity. However, the connectivity was higher at the end of both 1 and 6 cycles, indicating that the root system with a larger root diameter was not ideal for the crack-blocking effect.

From the indicators at the end of the 6th dry–wet cycle, it can be seen that the crack rate, average crack width, connectivity, and average crack length exhibited an overall trend of decreasing with a reduction in root diameter. The number of cracks exhibited an increase with decreasing root diameter. These results suggest that the crack-blocking effect of the root system is enhanced with a smaller root diameter and the best crack-blocking effect is achieved for a 0–0.4 mm root diameter. This phenomenon can be primarily attributed to the fact that under the same root-to-soil mass ratio conditions, roots with a smaller diameter in expansive soil tend to exhibit a greater number of roots, thereby exerting a more potent crack-blocking effect.

#### Crack development pattern

**Figure 13:**
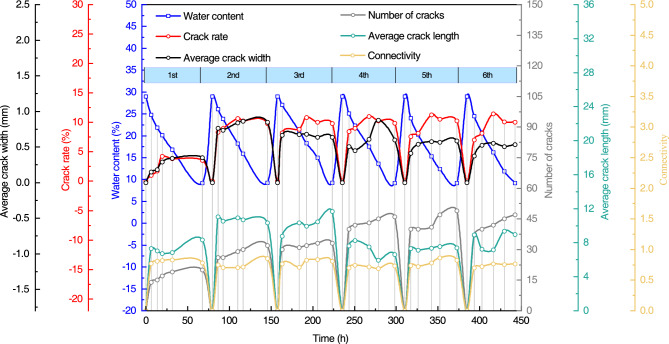
The development pattern of the crack index during the dry–wet cycles of root-doped expansive soil.

Figure 13 plots the development pattern of the cracks index of root-doped expansive soil (root-to-soil mass ratio of 0.14% and root diameter of 0–0.4 mm) during the dry–wet cycles (temperature 25 °C). The duration of the dehumidification process exhibited a gradual reduction with an increasing number of dry–wet cycles. This phenomenon can be primarily attributed to the progressive fragmentation of the soil as the dry–wet cycles progress, resulting in an increased crack area exposed to air and an accelerated rate of water evaporation. In the single dehumidification process, the dehumidification speed exhibited a decreasing trend. This can be mainly attributed to the faster evaporation of surface water from the soil compared to the water within the soil.

In the process of dehumidification, the change in the crack rate of the soil sample exhibited a rapid growth trend (between 30 and 15% water content), followed by a tendency to balance and a slight decrease (between 15 and 9% water content). This phenomenon is mainly due to the root system bridging the cracks generated by soil cracking and inhibiting further crack development. The average crack width exhibited a similar trend as the crack rate. This means that the main expression of the crack rate is the average crack width. Conversely, the trend of the average crack length was opposite to that of crack rate (between 20 and 15% water content), indicating that average crack length had little influence on crack rate. The connectivity and number of cracks in the soil samples exhibited an increasing trend. The average crack length exhibited a rapid increase in the early stage (soil samples with water content between 30 and 20%), followed by a slow decrease in the later stage (soil samples with water content between 20 and 9%). This can be attributed to the development of new cracks on top of the original long cracks in the later stage, resulting in the truncation of long cracks into shorter ones. This observation reflects a negative correlation between the number of cracks and the average crack length.

The microstructural characteristics of expansive soils during dry–wet cycles are important to explain the dry–wet cycle effects. Previous studies pointed out that the structural characteristics between the aggregates are the main factors determining the physical and mechanical properties of expansive soils^47^. In the process of water loss and shrinkage of expansive soil, the first thing consumed is the free water between the aggregates. As the suction of the soil increases, the effective stress of the agglomerates increases. As a result, the aggregates become closely aligned and even regroup together. However, this uneven shrinkage is very likely to promote soil cracking^48^. In the process of water absorption and expansion, the water absorbed by the expanded soil should first meet the demand of the aggregate binding water, and the water absorbed on this basis will be used for the filling of pores and cracks. As the suction of the soil decreases, the effective stress of the aggregate decreases. At this time, under the action of water wedging pressure and expansion pressure, the aggregates may be dispersed into the next level of aggregates. This increases the pore space between aggregates and makes them more loosely arranged^47,49,50^. The macroscopic manifestation is that the fractures will pseudo-heal with increasing water content. This also proves that the damage to the microstructure of the expansive soil is irreversible in terms of intergranular bonding during the dry–wet cycles.

### Effect of the root system on the strength of expansive soil

**Figure 14:**
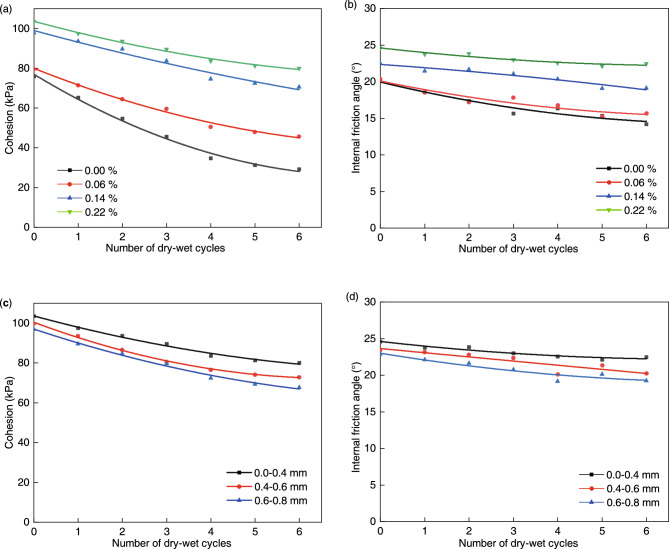
Influence of root-to-soil mass ratio (**a**)**–**(**b**) and root diameter (**c**)**–**(**d**) on shear strength index of expansive soil at the end of each dehumidification.

Figure 14 shows the cohesion and internal friction angle of the expansive soil as a function of the number of dry–wet cycles at the end of each dehumidification. The cohesion and internal friction angle of expansive soil with different root diameters and root-to-soil mass ratio exhibited a decreasing trend with the increase of dry–wet cycles, which indicated that the dry–wet cycles contributed to the decay of soil strength. This is because the dry–wet cycles promote the generation and expansion of cracks. The cracks in the soil destroy the structure of the soil, which directly reduces the shear strength of the soil on the one hand and increases the rate and depth of water infiltration on the other. The soil particles are filled with bound water film between them and the suction in the soil is gradually reduced. The decrease in suction reduces the effective stress between soil particles and thus the shear strength. Moreover, the cohesion and the internal friction angle increased with the increase of root-to-soil mass ratio and decreased with the increase of root diameter under the same number of dry–wet cycles.

Tables 4 and 5 show the results of the shear strength index of expansive soils with different root-to-soil mass ratios and root diameters at the end of the 0th and 6th dry–wet cycles respectively. The strength indexes of root-doped expansive soils were significantly higher than those of pure expansive soils before and after 6 dry–wet cycles. The cohesion and internal friction angle of the soils displayed an increasing trend with the increase of root-to-soil mass ratio and a decreasing trend with the increase of root diameter. This indicates that roots can improve the shear strength of soils, which is consistent with previous studies^51^. In addition, it was observed that shear strength decay showed a negative correlation with the root-to-soil mass ratio and a positive correlation with the root diameter. This phenomenon indicates that a higher root-soil mass ratio and smaller root diameter result in better inhibition of the shear strength decay. Vegetation with a well-developed root system, a large number of roots, and a small root diameter has significant advantages in expansive soil slope protection.

**Table 4:** Cohesion at the end of the 0th and 6th dry–wet cycles for different root-to-soil mass ratios and root diameters.

	Root-to-soil mass ratio				Root diameter		
	0.00%	0.06%	0.14%	0.22%	0–0.4 mm	0.4–0.6 mm	0.6–0.8 mm
N = 0, Cohesion (kPa)	75.89	79.68	97.85	103.56	103.56	99.85	96.85
N = 6, Cohesion (kPa)	29.16	45.68	70.58	79.98	79.98	72.89	67.65
Degradation (%)	61.6	42.7	27.9	22.8	22.8	27.0	30.1

**Table 5:** Internal friction angles of the 0th and 6th dry–wet cycles for different root-to-soil mass ratios and root diameters.

	Root-to-soil mass ratio				Root diameters		
	0.00%	0.06%	0.14%	0.22%	0–0.4 mm	0.4–0.6 mm	0.6–0.8 mm
N = 0, Internal friction angles (°)	20.1	20.4	22.5	24.6	24.6	23.45	22.86
N = 6, Internal friction angles (°)	14.2	15.68	19.12	22.45	22.45	20.25	19.25
Degradation (%)	29.4	23.1	15.0	8.7	8.7	13.6	15.8

### Relationship between crack development process and intensity decay

**Figure 15:**
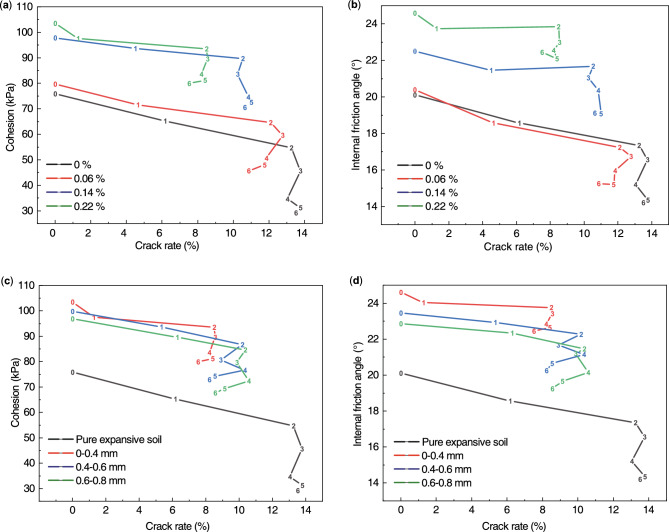
Relationship between the crack rate and soil shear strength index of expansive soils: (**a**)–(**b**) the influence of root-to-soil mass ratio; (**c**)–(**d**) the influence of root diameter; 0–6 the number of dry–wet cycles.

To reflect the relationship between crack and soil shear strength under dry–wet cyclic conditions, it was chosen to relate crack rate to the soil cohesion and the internal friction angle^28^. The relationship between the crack rate and soil shear strength index of expansive soils under the influence of root-to-soil mass ratio and root diameter was plotted as shown in Fig. 15.

As shown in Fig. 15(a)-(b), the crack rate of all groups of soil samples increased rapidly in the first two dry–wet cycles, followed by slight fluctuations, and eventually reached a relatively stable state. The expansive soil cohesion and internal friction angle exhibited a decreasing trend throughout the change of crack rate, and the value was stable in the 4th or 5th cycle. It can be observed that, for a given root diameter, the soil strength index during the dry–wet cycles exhibited a positive correlation with the root-soil mass ratio, while the increase in crack rate and the decrease in shear strength index exhibited a negative correlation with the root-soil mass ratio. It can be seen that the development of soil cracks was accompanied by a decrease in soil strength. Specifically, the cohesion of pure expansive soil decreased by 61.58% and the internal friction angle decreased by 29.36% after 6 dry–wet cycles. In contrast, the cohesive strength and the internal friction angle of expansive soil with a root-to-soil mass ratio of 0.22% only decreased by 22.77% and 8.73%, respectively, after the same number of dry–wet cycles.

The trend of the strength index of expansive soil under the influence of root diameter was similar to that under the influence of root-to-soil mass ratio. As shown in Fig. 15(c)-(d), the cohesion and the internal friction angle exhibited a decreasing trend throughout the change of the crack rate change and stabilized by the 5th cycle. It can be observed that, for a given root-to-soil mass ratio, the soil strength index during the dry–wet cycles exhibited a negative correlation with the root diameter, while the increase in crack rate and the decrease in shear strength index exhibited a positive correlation with the root diameter. This is mainly because the contact area between the root system and the soil increases with the increase in root diameter. In the pre-shrinkage stage of water loss in the expansive soil, the friction between the root system and the soil body can give full play to the crack-blocking effect of the root system and inhibit the expansion of cracks. However, with the evaporation of water, the soil particles close to each other, the larger the diameter of the root system and the larger the gap in the soil body. The friction between the root system and the soil will be reduced, and the root system will have a weaker crack-blocking effect.

## Root-enhanced soil expansion mechanism

**Figure 16:**
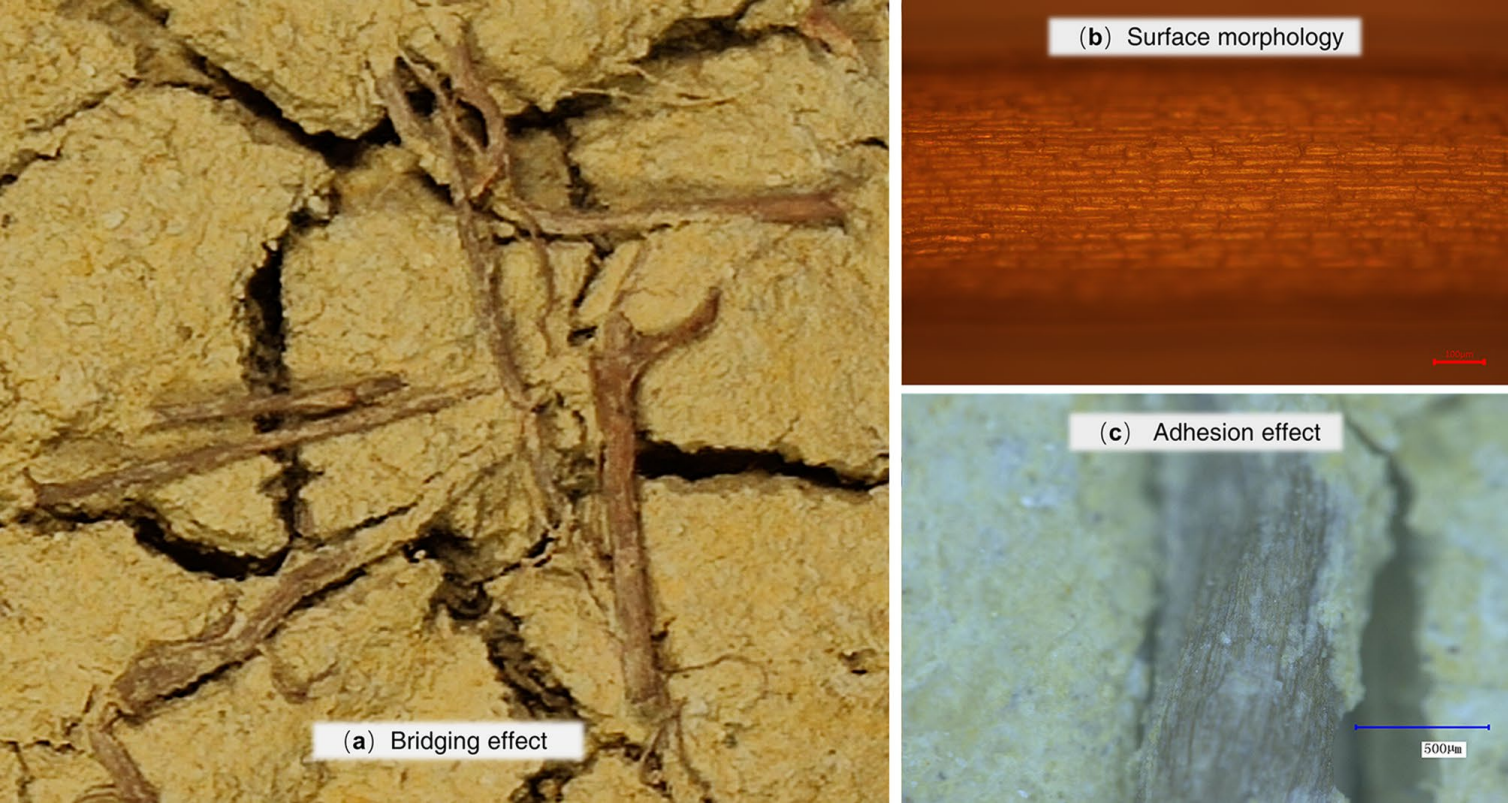
Root action on the soil and root surface characteristics: (**a**) bridging effect; (**b**) surface morphology; (**c**) adhesion effect.

Suction and tensile strength are critical mechanical parameters affecting the development of cracks in expansive soils, and the incorporation of Cynodon dactylon roots in expansive soils mainly affects the tensile strength of the soil during the shrinkage process. The root system of Cynodon dactylon roots forms a root-soil complex by becoming entangled and intertwined with the soil. During the dehumidification process, if the root system has sufficient tensile strength without crack, it will bridge the cracks and inhibit further crack development, as shown in Fig. 16(a). The bridging effect of the root system on the cracks results in a significant increase in the crack-blocking effects of the root-soil complex.

The root system is connected to the soil through the interface, and the root-soil composite can convert the shear stress in the soil into the tensile stress of the root system through the frictional bonding between the root and soil as well as the mechanical occlusion force^52^. This phenomenon helps to diffuse stress concentration in weak soil areas, resulting in more uniform strain distribution and delayed appearance of soil cracking surface, thus facilitating effective stress transfer. As shown in Fig. 16(b)-(c), the surface of the root system of Cynodon dactylon roots is rough and some of the soil particles will engage with each other with the root system^53^, which provides a better interfacial bond between the roots and the soil. Stronger interfacial bonding enables the root system to fully exert its mechanical properties and effectively enhance the overall mechanical properties of the root-soil composite.

## Conclusion

1. The cracks in the root-doped expansive soil are mostly shallow and fine disconnected cracks compared with the pure expansive soil after 6 dry–wet cycles, which proves that the Cynodon dactylon roots could significantly inhibit the development of cracks in the expansive soil.
2. The surface crack index of root-doped expansive soil exhibits significant phase characteristics during repeated dry–wet cycles, and the crack rate growth is expressed in the form of the development of the average crack width.
3. The crack-blocking and reinforcing effects on the root system become pronounced as the root-to-soil mass ratio increases and the root diameter decrease. When selecting the dominant vegetation in the expansive soil slope protection project, the vegetation with a well-developed root system, a large number of roots, and a small root diameter should be given priority.
4. The development of cracks in expansive soil is accompanied by a decrease in soil strength. The quantitative relationship between crack development and soil strength can be used to judge the strength and stability of slope soil, which plays a crucial role in the prevention and reinforcement of slope stability.

## Data Availability

The datasets generated and analyzed during the current study are not publicly available but are available from the corresponding author at reasonable request.

## References

[1] Mehta B, Sachan A (2017). Effect of mineralogical properties of expansive soil on its mechanical behavior. Geotech. Geol. Eng..

[2] Tang L, Cong SY, Geng L, Ling XZ, Gan F (2018). The effect of freeze-thaw cycling on the mechanical properties of expansive soils. Cold Reg. Sci. Technol..

[3] Hou TS (2013). Formation mechanism and stability analysis of the Houba expansive soil landslide. Eng. Geol..

[4] Qi SC, Vanapalli SK (2016). Influence of swelling behavior on the stability of an infinite unsaturated expansive soil slope. Comput. Geotech..

[5] Zhan TL, Ng CW, Fredlund DG (2007). Field study of rainfall infiltration into a grassed unsaturated expansive soil slope. Can. Geotech. J..

[6] Bi G (2023). A preliminary study of the application of the strain-self-sensing smart geogrid rib in expansive soils. Geotext. Geomembr..

[7] Prambauer M, Wendeler C, Weitzenböck J, Burgstaller C (2019). Biodegradable geotextiles–an overview of existing and potential materials. Geotext. Geomembr..

[8] Xu YF, Zhang HR (2021). Design of soilbag-protected slopes in expansive soils. Geotext. Geomembr..

[9] Yang T, Zou JF, Pan QJ (2020). Three-dimensional seismic stability of slopes reinforced by soil nails. Comput. Geotech..

[10] Chaduvula U, Viswanadham BVS, Kodikara J (2022). Centrifuge model studies on desiccation cracking behaviour of fiber-reinforced expansive clay. Geotext. Geomembr..

[11] Huang Z (2022). A study on the shear strength and dry-wet cracking behaviour of waste fibre-reinforced expansive soil. Case Stud. Constr. Mater..

[12] Wang YX (2017). Laboratory investigation on strength characteristics of expansive soil treated with jute fiber reinforcement. Int. J. Geomech..

[13] Emarah DA, Seleem SA (2018). Swelling soils treatment using lime and sea water for roads construction. Alex. Eng. J..

[14] Sahoo JP, Pradhan PK (2010). Effect of lime stabilized soil cushion on strength behaviour of expansive soil. Geotech. Geol. Eng..

[15] Tiwari N, Satyam N (2020). An experimental study on the behavior of lime and silica fume treated coir geotextile reinforced expansive soil subgrade. Eng. Sci. Technol. Int. J..

[16] Lu Y (2021). Impact of biochar on the desiccation cracking behavior of silty clay and its mechanisms. Sci. Total Environ..

[17] Santhikala R, Chandramouli K, Pannirselvam N (2022). Stabilization of expansive soil using flyash based geopolymer. Mater. Today Proc..

[18] Xu YZ, Su C, Huang Z, Yang CY, Yang YH (2022). Research on the protection of expansive soil slopes under heavy rainfall by anchor-reinforced vegetation systems. Geotext. Geomembr..

[19] Fu HY (2020). Research progress on ecological protection technology of highway slope: Status and challenges. Transp. Saf. Environ..

[20] Colombi T (2021). A time-lapse imaging platform for quantification of soil crack development due to simulated root water uptake. Soil Tillage Res..

[21] Gyssels G, Poesen J, Bochet E, Li Y (2005). Impact of plant roots on the resistance of soils to erosion by water: A review. Prog. Phys. Geogr..

[22] Li JH, Li L, Chen R, Li DQ (2016). Cracking and vertical preferential flow through landfill clay liners. Eng. Geol..

[23] Wang C (2021). Geometrical and statistical analysis of dynamic crack morphology in shrink-swell soils with addition of maize roots or salinity (NaCl). Soil Tillage Res..

[24] Wang GY, Huang YG, Li RF, Chang JM, Fu JL (2020). Influence of vetiver root system on mechanical performance of expansive soil: Experimental studies. Adv. Civ. Eng..

[25] Bordoloi S, Ng CWW (2020). The effects of vegetation traits and their stability functions in bio-engineered slopes: A perspective review. Eng. Geol..

[26] Xie CR, Ni PP, Xu MJ, Mei GX, Zhao YL (2020). Combined measure of geometry optimization and vegetation for expansive soil slopes. Comput. Geotech..

[27] Danxi S (2023). Three-dimensional characterization of cracks in undisturbed Mile expansive soil using X-ray computed tomography. Soils Found..

[28] Huang Z, Wei BX, Zhang LJ, Chen W, Peng ZM (2019). Surface crack development rules and shear strength of compacted expansive soil due to dry–wet cycles. Geotech. Geol. Eng..

[29] Khan MS, Hossain S, Ahmed A, Faysal M (2017). Investigation of a shallow slope failure on expansive clay in Texas. Eng. Geol..

[30] Li JH, Zhang LM (2011). Study of desiccation crack initiation and development at ground surface. Eng. Geol..

[31] Zhao GT, Zou WL, Han Z, Wang DX, Wang XQ (2021). Evolution of soil-water and shrinkage characteristics of an expansive clay during freeze-thaw and drying-wetting cycles. Cold Reg. Sci. Technol..

[32] Zhu R, Huang YH, Zhang C, Guo WL, Chen H (2022). Laboratory and centrifugal model tests on failure mechanism of canal slopes under cyclic action of wetting–drying. Eur. J. Environ. Civ. Eng..

[33] Amiri E, Emami H, Mosaddeghi MR, Astaraei AR (2019). Shear strength of an unsaturated loam soil as affected by vetiver and polyacrylamide. Soil Tillage Res..

[34] Bordoloi S, Ni J, Ng CWW (2020). Soil desiccation cracking and its characterization in vegetated soil: A perspective review. Sci. Total Environ..

[35] Comino E, Marengo P, Rolli V (2010). Root reinforcement effect of different grass species: A comparison between experimental and models results. Soil Tillage Res..

[36] Ji, X. L. GDS triaxial test on the reinforcement effects of bermudagrass root-soil complex. In *IOP Conference Series: Earth and Environmental Science* (Vol. 304, No. 3, p. 032106). IOP Publishing. 10.1088/1755-1315/304/3/032106 (2019).

[37] Ye C, Li SY, Zhang YL, Tong XZ, Zhang QF (2013). Assessing heavy metal pollution in the water level fluctuation zone of China’s Three Gorges Reservoir using geochemical and soil microbial approaches. Environ. Monit. Assess..

[38] Cheng L, Hao YZ (2018). Experimental study on the root characteristic parameters impact on soil strength. Sci. Tech. Eng..

[39] Xu WX (2019). Impacts of the typical herbaceous plant roots on soil scour resistance in the reservoir riparian zone. J. Soil Water Conserv..

[40] Xian SH (2016). Research on the Protective Effect of Anchor Reinforced Vegetation System on Expansive Soil Slope Surface.

[41] Zhao ZY, Wang SJ, Yang ZB (2020). Quantitative analysis of fracture evolution of expansive soils under wetting-drying cycles. Rock Soil Mech..

[42] Liu C, Shi B, Zhou J, Tang C (2011). Quantification and characterization of microporosity by image processing, geometric measurement and statistical methods: Application on SEM images of clay materials. Appl. Clay Sci..

[43] Liu C, Tang CS, Shi B, Suo WB (2013). Automatic quantification of crack patterns by image processing. Comput. Geosci..

[44] Tang CS, Cui YJ, Tang AM, Shi B (2011). Volumetric shrinkage characteristics of soil during drying. Chin. J. Geotech. Eng..

[45] Tang CS, Shi B, Liu C, Suo WB, Gao L (2011). Experimental characterization of shrinkage and desiccation cracking in thin clay layer. Appl. Clay Sci..

[46] Tang CS, Wang DY, Shi B, Liu C (2013). Quantitative analysis of soil desiccation crack network. Chin. J. Geotech. Eng..

[47] Wu K (2016). Swelling-shrinking characteristics and irreversible deformation of expansive soil during wetting-drying cycles. J. Harbin Inst. Technol..

[48] Tang CS, Shi B, Liu C (2012). Study on desiccation cracking behaviour of expansive soil. J. Eng. Geol..

[49] Zemenu G, Martine A, Roger C (2009). Analysis of the behaviour of a natural expansive soil under cyclic drying and wetting. Bull. Eng. Geol. Environ..

[50] Lü HB, Zeng ZT, Zhao YL, Lu H (2009). Experimental studies of strength of expansive soil in drying and wetting cycle. Rock Soil Mech..

[51] Waldron LJ (1977). The shear resistance of root-permeated homogeneous and stratified soil. Soil Sci. Soc. Am. J..

[52] Wang Y (2022). Fragmentation of soil-root complexes in sloping landscapes during tillage and soil translocation. Biosys. Eng..

[53] De Baets S (2008). Root tensile strength and root distribution of typical Mediterranean plant species and their contribution to soil shear strength. Plant Soil.

